# Comparative value of retinal structural features versus systemic inflammatory and renal indicators in predicting short-term anti-VEGF response in diabetic macular edema: focus on OCT biomarkers

**DOI:** 10.3389/fmed.2026.1761324

**Published:** 2026-02-03

**Authors:** Xin Li, Lan Yang, He Long, Shaomin Peng

**Affiliations:** 1 Aier Eye Medical Center of Anhui Medical University, Hefei, Anhui, China; 2 Wuhan Aier Eye Hanyang Hospital, Wuhan, China; 3 Harbin Aier Eye Hospital, Harbin, China

**Keywords:** anti-VEGF therapy, diabetic macular edema, optical coherence tomography, predictive model, retinal structure, treatment response

## Abstract

**Objective:**

This study aimed to systemically compare the predictive value of systemic inflammatory indicators, renal function, and optical coherence tomography (OCT)-derived morphological characteristics for the short-term response to anti-vascular endothelial growth factor (anti-VEGF) therapy in patients with diabetic macular edema (DME), and to clarify their relative importance.

**Methods:**

A single-center retrospective observational study was conducted involving 81 DME patients who completed three monthly loading doses of anti-VEGF therapy. Baseline data including systemic inflammatory indicators (neutrophil-to-lymphocyte ratio [NLR], systemic immune-inflammatory index [SII], platelet-to-lymphocyte ratio [PLR]), renal function (estimated glomerular filtration rate [eGFR]), and comprehensive OCT morphological features were collected. Patients were categorized into a poor-response group (*n* = 28) and a response group (*n* = 53) based on an improvement in best-corrected visual acuity (BCVA) of ≤5 letters at 3 months. Multivariate logistic regression analysis was employed to identify independent predictive factors. A predictive model was constructed and evaluated for its discrimination (using AUC), calibration, and clinical utility.

**Results:**

Univariate analysis identified eGFR, and several OCT features as significant predictors eligible for the multivariate model, whereas none of the systemic inflammatory indicators (NLR, SII, PLR) showed significant predictive value. Multivariate analysis revealed that specific OCT structural features, namely disorganization of inner retinal layers (DIRT) (OR = 0.093, 95% CI [0.021–0.412], *p* = 0.002) and disruption of the ellipsoid zone/external limiting membrane (EZ/ELM) (OR = 0.142, 95% CI [0.032–0.628], *p* = 0.010), were strong independent predictors of poor treatment response. A predictive model integrating eGFR and these two OCT features demonstrated excellent discrimination, with an area under the curve (AUC) of 0.869 in the training set and a bootstrap-validated average AUC of 0.887. At the optimal cutoff value, the model achieved a specificity of 0.893, indicating a strong capability to identify high-risk patients.

**Conclusion:**

Our comparative analysis confirms that baseline retinal structural integrity assessed by OCT—specifically disorganization of the inner retinal layers and disruption of the EZ/ELM—shows stronger predictive value for short-term anti-VEGF response in DME compared to systemic inflammatory indicators. The developed prediction model demonstrates good discriminative performance, though its clinical application requires further validation in larger cohorts. These findings support the prioritization of OCT imaging in DME management.

## 1 Introduction

Diabetic macular edema (DME) stands as a leading cause of vision impairment among patients with diabetes, with its pathological core revolving around the breakdown of the blood-retinal barrier. Intravitreal injection of anti-vascular endothelial growth factor (anti-VEGF) agents has emerged as a first-line treatment for DME, effectively reducing macular edema and improving visual acuity (1, 2). However, significant interindividual variation exists in clinical practice, with approximately one-third of patients exhibiting a suboptimal response to anti-VEGF therapy. This constitutes a central challenge in the current management of DME (3, 4).

To identify potential poor-responders early, various biomarkers have been explored for their predictive value. On one hand, systemic biomarkers derived from blood tests have garnered significant interest. Systemic inflammation is recognized as a key pathogenetic mechanism in DME, leading to the hypothesis that systemic inflammatory indices, such as the neutrophil-to-lymphocyte ratio (NLR), systemic immune-inflammatory index (SII), and platelet-to-lymphocyte ratio (PLR), could serve as potential predictive factors (5). Furthermore, renal insufficiency, a systemic manifestation of diabetic microvascular complications, has also been investigated for its predictive potential, with the estimated glomerular filtration rate (eGFR) being a representative indicator (6). On the other hand, advancements in imaging technology have revealed that microstructural characteristics of the retina identified by optical coherence tomography (OCT), such as disorganization of the inner retinal layers (DIRT) and disruption of the ellipsoid zone/external limiting membrane (EZ/ELM), show considerable promise in assessing disease severity and forecasting prognosis (7).

Although the systemic indicators mentioned above have shown some predictive potential, the recent research focus has gradually shifted toward optical coherence tomography (OCT) features that can more directly reflect the local pathological status of the macula. Several studies have confirmed that specific OCT morphological changes, such as DIRT and EZ/ELM disruption, are closely associated with the severity of DME and the response to anti-VEGF therapy (8, 9). Consequently, a key scientific question that remains insufficiently addressed is: When predicting the response to anti-VEGF therapy, do these local OCT features, which directly reflect the focal pathology, hold superior predictive value compared to systemic inflammatory indices that are susceptible to the systemic condition? Elucidating this “relative importance” is crucial for clinicians to prioritize the most direct and stable tools for clinical decision-making amidst a multitude of available indicators. Currently, few studies have systematically and directly compared and validated these two distinct categories of predictors within the same cohort.

Based on this rationale, this study aims to systematically compare and evaluate the relative predictive power of systemic inflammatory indicators and OCT structural features for the response to anti-VEGF therapy in patients with DME, utilizing a retrospective cohort design. The core objective of our research is not to negate previous findings but to further clarify the hierarchical relationship among different categories of predictors within a unified statistical framework. We seek to identify genuine independent predictive factors through multivariate modeling and to construct a comprehensive prediction model. The novelty of this study lies in its aim to move beyond reporting the mere presence or absence of value in individual indicators. Instead, through direct comparison, it aims to provide high-level evidence-based support for establishing a more clinically operable prognostic assessment system centered on objective local imaging evidence.

## 2 Materials and methods

### 2.1 Study design and ethical approval

This was a single-center, retrospective, observational study conducted in accordance with the tenets of the Declaration of Helsinki. The study protocol was reviewed and approved by the Ethics Committee of Wuhan Aier Eye Hanyang Hospital (Approval No. IEC-AD-HYEYE2024008), which waived the requirement for informed consent due to the retrospective nature of the research.

### 2.2 Study participants

The study collected medical records of patients diagnosed with DME who received intravitreal anti-VEGF injections at our hospital between July 2023 and July 2025. For patients with bilateral disease, the right eye was selected for study inclusion; in cases of unilateral involvement, the affected eye was enrolled.

Inclusion criteria were as follows: (1) confirmed diagnosis of DME; (2) treatment-naïve and completion of three consecutive monthly intravitreal injections of aflibercept (Eylea, Bayer AG); (3) availability of complete baseline clinical data, laboratory test results, and high-quality OCT images; and (4) complete follow-up data, including OCT and BCVA measurements, at 1 month after the third injection.

Exclusion criteria included: (1) comorbid ocular conditions that could cause macular edema (e.g., retinal vein occlusion, age-related macular degeneration); (2) history of intraocular surgery (e.g., vitrectomy); (3) significant media opacities (corneal, lens, or vitreous) affecting OCT image quality; and (4) incomplete follow-up data.

### 2.3 Treatment response assessment and grouping

The operational definition of “poor response” was clearly specified. Patients were classified into a “poor-response group” and a “response group” based on whether the improvement in Best-Corrected Visual Acuity (BCVA), measured in ETDRS letters, was ≤5 letters at the one-month follow-up visit after the completion of the three loading doses.

### 2.4 Data collection

The following baseline data were systematically collected:

Basic clinical characteristics: Age, gender, glycated hemoglobin (HbA1c), history of hypertension, baseline BCVA, and central subfield thickness (CST).
Hematological indices: Baseline neutrophil count, lymphocyte count, and platelet count were obtained to calculate systemic inflammatory indices, including the neutrophil-to-lymphocyte ratio (NLR), platelet-to-lymphocyte ratio (PLR), and systemic immune-inflammation index (SII).
Renal function parameter: Serum creatinine level was measured, and the estimated glomerular filtration rate (eGFR) was calculated using the Chronic Kidney Disease Epidemiology Collaboration (CKD-EPI) formula based on serum creatinine, age, and sex.

### 2.5 OCT image analysis

Qualitative analysis of baseline OCT images was performed using the Zeiss CIRRUS HD-OCT 5000 device. The analysis was conducted independently by two retinal specialists who were blinded to the patients’ treatment response and group assignment. Discrepancies were resolved through discussion until a consensus was reached. The following morphological features were recorded: subretinal fluid (SRF); large intraretinal cysts (IRC), defined as the presence of round, hyporeflective spaces >250 μm in diameter within the 750 μm central foveal area; disorganization of the inner retinal layers (DIRT); integrity of the ellipsoid zone/external limiting membrane (EZ/ELM); and presence of hyperreflective dots (HRDs), defined as the presence of a total of more than 30 hyperreflective dots across all sections of the macular radial scans.

### 2.6 Statistical analysis

All statistical analyses were performed using Python (version 3.13). Data manipulation was conducted with the Pandas library. Statistical tests were carried out using Scipy, logistic regression modeling was performed with Statsmodels, and data visualization was achieved using Matplotlib and Seaborn. Continuous variables following a normal distribution were presented as mean ± standard deviation and compared between groups using the independent samples *t*-test (scipy. stats. ttest_ind). Non-normally distributed continuous data were expressed as median (interquartile range) and compared using the Mann–Whitney U test (scipy. stats. mannwhitneyu). Categorical variables were presented as numbers (percentages) and compared using the Chi-square test (scipy. stats. chi2_contingency).

Variables with a *p*-value <0.2 in the univariate analysis were included in the multivariate logistic regression model (statsmodels. api. Logit). Given the exploratory nature of this study and sample size considerations, stepwise regression was employed for variable selection, while acknowledging its limitations in identifying clinically relevant variables. eGFR was retained in the final model based on its significance in univariate analysis (*p* = 0.020) and clinical relevance to diabetic microvascular complications, despite not reaching independent significance in multivariate analysis.

Based on the results of the multivariate logistic regression, a nomogram prediction model was constructed using the Matplotlib library for visualization. The discriminative ability of the model was evaluated by the area under the receiver operating characteristic curve (AUC). The optimal cutoff value (0.737) was determined using the Youden index from the ROC curve to balance sensitivity and specificity. Calibration was assessed using calibration curves (with 1,000 bootstrap resamples) and the Hosmer-Lemeshow test. Decision curve analysis (DCA) was applied to evaluate the clinical utility of the model. A two-sided *p*-value <0.05 was considered statistically significant.

## 3 Results

### 3.1 Comparison of baseline characteristics

A total of 81 patients were included in this study, with 53 in the responder group and 28 in the poor-responder group. The comparison of baseline characteristics between the two groups is presented in Table 1. No significant differences were observed between the groups in demographic indicators, including age 57.45 ± 11.62 years in the responder group vs. 57.89 ± 9.07 years in the poor-responder group (*p* = 0.515), and gender distribution (female/male: 19/34 vs. 14/14, *p* = 0.320). Regarding hematological parameters, no statistically significant differences were found between the groups for neutrophil count, lymphocyte count, platelet count, NLR, SII, or PLR (all *p* > 0.05). For renal function indicators, eGFR was significantly higher in the responder group than in the poor-responder group (82.06 ± 28.10 mL/min/1.73m^2^ vs. 67.05 ± 24.55 mL/min/1.73m^2^, *p* = 0.020). Concerning ophthalmic parameters, baseline ETDRS letters and baseline CST did not differ significantly between groups. However, the ETDRS letter score at 1 month after completing the three loading doses was significantly higher in the responder group (60.74 ± 15.38 vs. 49.36 ± 18.54, *p* = 0.004). Among metabolic parameters, HbA1c showed no significant difference between groups (*p* = 0.076). The history of hypertension also did not differ significantly (*p* = 0.313).

**Table 1:** Comparison of baseline characteristics between responder and poor-responder groups.

Variables	Responder group (*n* = 53)	Poor-responder group (*n* = 28)	Test	Statistic	*p* value	Effect size (*d*)
Demographics						
Age (years)	57.45 ± 11.62	57.89 ± 9.07	MWU	676.000	0.515	−0.072
Gender (female/male)	19/34	14/14	x^2^-test	0.990	0.320	0.111
Hematological parameters						
Neutrophil count (×10^9^/L)	4.07 ± 1.05	3.83 ± 0.86	*t*-test	−1.055	0.295	−0.246
Lymphocyte count (×10^9^/L)	1.67 ± 0.56	1.54 ± 0.53	MWU	654.000	0.385	−0.097
Platelet count (×10^9^/L)	233.54 ± 53.45	215.39 ± 33.08	Welch’s t	−1.882	0.064	−0.382
NLR	2.63 ± 0.92	2.68 ± 1.35	MWU	716.000	0.800	0.044
SII	611.20 ± 259.51	560.26 ± 260.96	MWU	670.000	0.478	−0.196
PLR	154.20 ± 64.86	151.73 ± 44.07	MWU	816.500	0.648	−0.120
Renal function						
eGFR (mL/min/1.73m^2^)	82.06 ± 28.10	67.05 ± 24.55	t-test	−2.384	0.020	−0.557
Ophthalmic parameters						
Baseline EDTRS letters	45.99 ± 16.25	49.72 ± 17.82	MWU	655.000	0.388	0.222
Post-treatment EDTRS letters	60.74 ± 15.38	49.36 ± 18.54	t-test	2.947	0.004	−0.689
Baseline CST (mean)	304.16 ± 165.96	306.02 ± 207.67	MWU	775.500	0.743	0.010
CST (mean) at 1 month after 3 injections	191.02 ± 73.40	220.66 ± 94.42	MWU	581.000	0.111	0.365
Metabolic parameters						
HbA1c (%)	5.90 ± 1.61	6.42 ± 1.59	MWU	563.000	0.076	0.325
Medical history						
History of hypertension (No/Yes)	11/42	8/20	Fisher	-	0.313	−0.164

MWU, Mann–Whitney U test; x^2^-test, Chi-square test; Fisher, Fisher’s exact test. Data presented as mean ± standard deviation or *n* (%). *p*-values from appropriate statistical tests. Effect sizes (Cohen’s d) provided for continuous variables.

### 3.2 Prevalence of OCT indicators

The prevalence rates of OCT indicators in the responder and poor-responder groups are compared in Table 2. Disorganization of the inner retinal layers (35.8% vs. 89.3%, *p* < 0.001), disruption of the ellipsoid zone/external limiting membrane integrity (7.5% vs. 50.0%, *p* < 0.001), and hyperreflective dots (26.4% vs. 57.1%, *p* = 0.013) were significantly more common in the poor-responder group. In contrast, the presence of intraretinal cysts (*p* = 0.221) and subretinal fluid (*p* = 0.876) did not differ significantly between the two groups.

**Table 2:** Prevalence of OCT indicators in response groups.

OCT indicators	Response group (*n* = 53)	Poor-response group (*n* = 28)	Statistic	*P*-value
Inner retinal structure disorder	19 (35.8%)	25 (89.3%)	18.985	<0.001
Ellipsoid zone/external limiting membrane integrity disruption	4 (7.5%)	14 (50.0%)	16.7265	<0.001
Hyperreflective dots	14 (26.4%)	16 (57.1%)	6.1589	0.013
Intraretinal cysts	29 (54.7%)	20 (71.4%)	1.4988	0.221
Subretinal fluid	11 (20.8%)	7 (25.0%)	0.0244	0.876

### 3.3 Univariate regression analysis

Univariate linear regression analyses were performed to assess the influence of various variables on the percentage reduction in CST and improvement in BCVA. As shown in Table 3, for the percentage reduction in CST, the regression coefficients for NLR, SII, PLR, age, HbA1c, and eGFR were not statistically significant (all *p* > 0.05). The low adjusted *R*^2^ values indicated limited explanatory power of these individual variables for CST reduction.

**Table 3:** Univariate linear regression analysis for CST reduction percentage.

Independent variable	Coefficient (*β*)	Standard error	*P*-value	95% confidence interval	Adjusted *R*^2^
NLR	3.1229	3.20	0.3301	[−3.2204, 9.4662]	−0.0005
SII	0.0110	0.013	0.4118	[−0.0155, 0.0374]	−0.0040
PLR	−0.0622	0.057	0.2761	[−0.1750, 0.0507]	0.0025
Age	−0.4321	0.318	0.1785	[−1.0657, 0.2015]	0.0104
HbA1c (%)	0.8183	2.15	0.7038	[−3.4507, 5.0872]	−0.0108
eGFR	−0.0033	0.125	0.9788	[−0.2519, 0.2453]	−0.0126

CST, central subfield thickness; NLR, neutrophil-to-lymphocyte ratio; SII, systemic immune-inflammation index; PLR, platelet-to-lymphocyte ratio; HbA1c, glycated hemoglobin; eGFR, estimated glomerular filtration rate.

Similarly, as shown in Table 4, for BCVA improvement (in letters), the regression coefficients for NLR, SII, PLR, age, HbA1c, and eGFR were also not significant (all *p* > 0.05). The adjusted *R*^2^ values were close to zero, suggesting no significant linear relationship between these variables and BCVA improvement.

**Table 4:** Univariate linear regression analysis for BCVA improvement in letters.

Independent variable	Coefficient (*β*)	Standard error	*P*-value	95% confidence interval	Adjusted *R*^2^
NLR	0.9072	1.13	0.4248	[−1.3436, 3.1581]	−0.0045
SII	0.0045	0.005	0.3425	[−0.0049, 0.0138]	−0.0011
PLR	−0.0069	0.020	0.7349	[−0.0471, 0.0334]	−0.0112
Age	−0.0448	0.114	0.6954	[−0.2715, 0.1820]	−0.0107
HbA1c (%)	−0.5728	0.758	0.4517	[−2.0806, 0.9349]	−0.0054
eGFR	0.0502	0.044	0.2558	[−0.0371, 0.1375]	0.0039

BCVA, best-corrected visual acuity; other abbreviations as in Table 3.

### 3.4 Multivariable regression analysis

Multivariable regression analyses were conducted to evaluate the independent effects of multiple variables on treatment response, BCVA improvement, and CST reduction; the results are shown in Table 5. For treatment response (logistic regression), disorganization of the inner retinal layers (OR = 0.093, *p* = 0.002) and EZ/ELM integrity disruption (OR = 0.142, *p* = 0.010) were independent predictors of poor treatment response, while HbA1c, eGFR, intraretinal cysts, and hyperreflective dots showed no significant influence. For BCVA improvement (linear regression), EZ/ELM integrity disruption was negatively associated with BCVA improvement (*β* = −5.867, *p* = 0.044), whereas subretinal fluid and inner retinal structure disorder showed borderline significance (*p* < 0.1). For CST reduction (linear regression), intraretinal cysts were positively associated with CST reduction (*β* = 17.160, *p* = 0.013), while age had no significant effect.

**Table 5:** Multivariable regression analysis of treatment response, BCVA improvement, and central subfield thickness reduction.

Variable	Model	Coefficient	OR	OR_CI_Lower	OR_CI_Upper	*P*-value
HbA1c (%)	Treatment response (logistic)	0.022207102	1.022455515	0.69376621	1.5068697	0.910640929
eGFR (mL/min/1.73m^2^)	Treatment response (logistic)	0.020320152	1.020528011	0.994696131	1.047030736	0.120316278
Inner retinal structure disorder	Treatment response (logistic)	−2.374204142	0.093088545	0.020002339	0.433223204	0.002476237
Intraretinal cysts (IRC)	Treatment response (logistic)	−0.260080036	0.770989876	0.201110511	2.955714581	0.704439814
Hyperreflective dots (HRDs)	Treatment response (logistic)	−1.051389191	0.349451956	0.094188593	1.296512301	0.115999119
EZ/ELM integrity disruption	Treatment response (logistic)	−1.952975529	0.14185136	0.032286201	0.623232461	0.009706069
Subretinal fluid (SRF)	BCVA improvement (linear)	−5.639918107	N/A	N/A	N/A	0.065062229
Inner retinal structure disorder	BCVA improvement (linear)	−4.19315455	N/A	N/A	N/A	0.081391356
Hyperreflective dots (HRDs)	BCVA improvement (linear)	−2.036667946	N/A	N/A	N/A	0.452273399
EZ/ELM integrity disruption	BCVA improvement (linear)	−5.866923994	N/A	N/A	N/A	0.044333195
Age (years)	CST reduction (linear)	−0.393835287	N/A	N/A	N/A	0.205041689
Intraretinal cysts (IRC)	CST reduction (linear)	17.16042785	N/A	N/A	N/A	0.012796956

**Figure 1:**
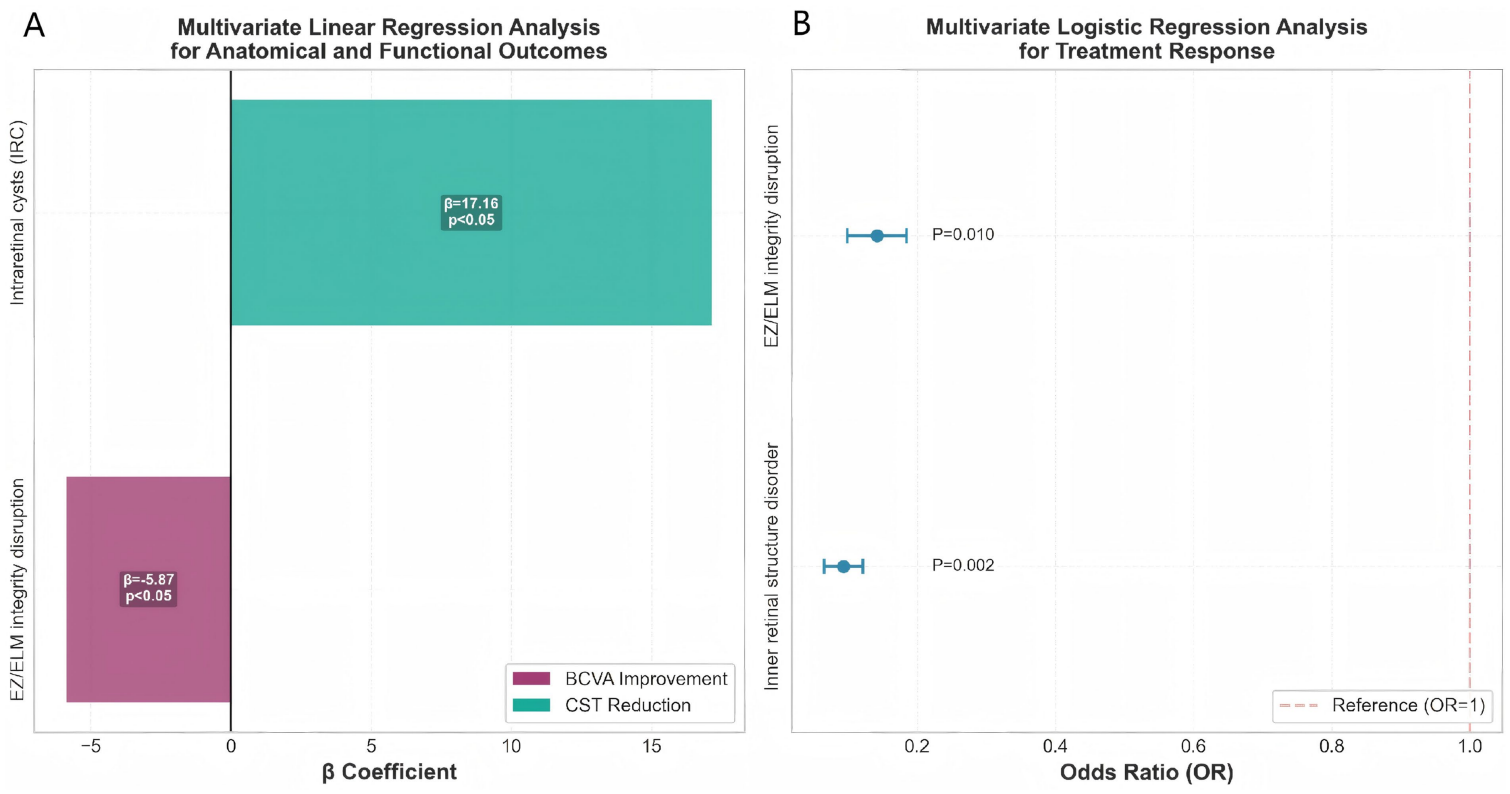
Multivariate regression analysis of treatment response predictors. **(A)** Multivariate linear regression analysis for anatomical and functional outcomes. **(B)** Multivariate logistic regression analysis for treatment response.

The results of the multivariable analysis were further visualized in Figures 1A,B. Figure 1A shows the linear regression coefficients for anatomical and functional outcomes: EZ/ELM integrity disruption had a negative effect on BCVA improvement (*β* = −5.867, *p* = 0.044), while intraretinal cysts had a positive effect on CST reduction (β = 17.160, *p* = 0.013). Figure 1B presents the odds ratios for the treatment response model in a forest plot, confirming that disorganization of the inner retinal layers (OR = 0.093, *p* = 0.002) and EZ/ELM integrity disruption (OR = 0.142, *p* = 0.010) were significant predictors.

### 3.5 Prediction model construction and performance

Based on variables screened by univariate analysis and clinical significance, four predictor variables were ultimately included in the model: eGFR, disorganization of the inner retinal layers, EZ/ELM integrity disruption, and hyperreflective dots. The model coefficients and odds ratios are shown in Table 6. The OR for eGFR was 1.022 (95% CI: 0.996–1.048), indicating it was a positive, though not statistically significant, factor for treatment response. The three OCT features were all significant negative predictors, with disorganization of the inner retinal layers (OR = 0.091, 95% CI: 0.021–0.389) and EZ/ELM integrity disruption (OR = 0.143, 95% CI: 0.033–0.617) showing the strongest predictive effects. Hyperreflective dots (OR = 0.354, 95% CI: 0.101–1.244) also showed a negative predictive trend.

**Table 6:** Coefficients and odds ratios of the prediction model variables.

Variable	Coefficient	Odds ratio (OR)	95% confidence interval
Intercept	1.5468	4.696	0.556–39.659
eGFR	0.0216	1.022	0.996–1.048
Inner retinal structure disorder	−2.3977	0.091	0.021–0.389
EZ/ELM integrity	−1.9473	0.143	0.033–0.617
Hyperreflective dots (HRDs)	−1.0388	0.354	0.101–1.244

The classification performance of the model at the optimal threshold (0.737) is shown in Table 7. The accuracy was 0.827, sensitivity was 0.792, specificity was 0.893, positive predictive value was 0.933, and negative predictive value was 0.694, indicating good discriminative ability. To evaluate the model’s generalizability, internal validation was performed using 1,000 bootstrap samples. The validation results showed a mean AUC of 0.887 (95% confidence interval CI: 0.791–0.958), a mean sensitivity of 0.885 (95% CI: 0.767–0.969), and a mean specificity of 0.711 (95% CI: 0.440–0.926). These results suggest the model has robust predictive performance.

**Table 7:** Classification performance of the model at the optimal threshold (0.737).

Performance metric	Value
Accuracy	0.827
Sensitivity	0.792
Specificity	0.893
Positive predictive value	0.933
Negative predictive value	0.694

**Figure 2:**
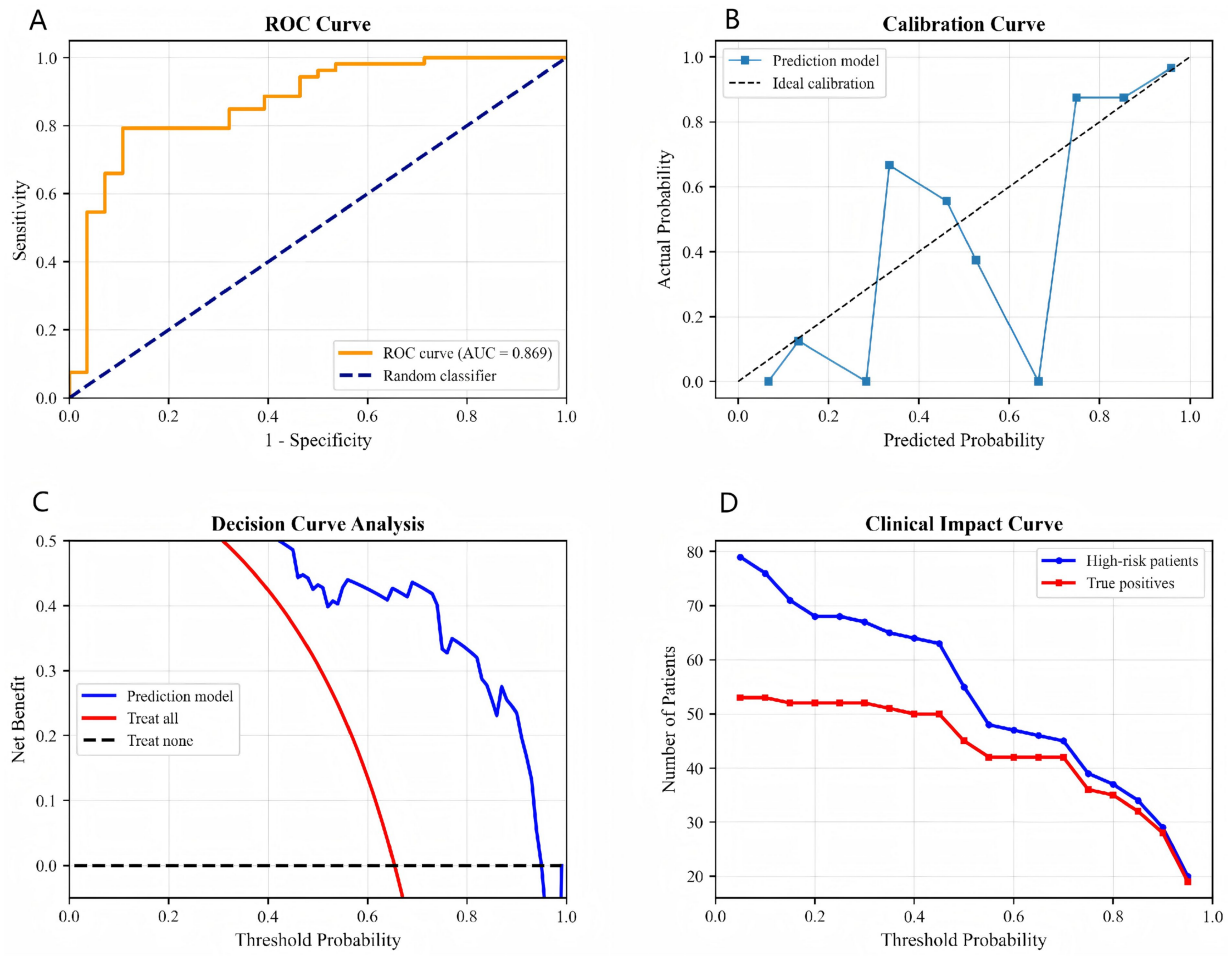
Comprehensive model validation: **(A)** ROC curve, **(B)** calibration curve, **(C)** decision curve analysis, **(D)** clinical impact curve.

Model performance was further evaluated using the ROC curve, calibration curve, decision curve analysis, and clinical impact curve (Figures 2A–D). The ROC curve in Figure 2A shows an AUC of 0.869, indicating excellent discriminatory ability. The calibration curve in Figure 2B shows that the predicted probabilities generally aligned with the actual probabilities, albeit with slight deviation. The decision curve analysis in Figure 2C indicates that using the model provided a net clinical benefit within the threshold probability range of 0.2–0.8. The clinical impact curve in Figure 2D shows the distribution of high-risk patients and true positive cases at different thresholds, providing a basis for resource allocation.

## 4 Discussion

Our findings appear to provide evidence that retinal microstructural features assessed by OCT may serve as potentially useful predictors of short-term anti-VEGF response in DME when compared to systemic inflammatory and renal indicators. These observations seem to align with previous studies that have suggested the prognostic value of specific OCT biomarkers (7–10). The comparative framework used in our study attempts to simultaneously evaluate both systemic and local predictors within a unified multivariate model, which may offer a more balanced perspective on the relative importance of different predictor categories in DME management.

The observed better predictive performance of OCT-derived features, particularly DIRT and EZ/ELM disruption, might underscore the potential importance of assessing end-organ damage in addition to systemic surrogate markers. This finding could have implications for clinical practice, suggesting that retinal specialists might consider prioritizing high-resolution imaging biomarkers when making treatment decisions, while still acknowledging the value of comprehensive patient assessment.

The predictive value observed for DIRT and EZ/ELM disruption might be explained by their potential reflection of structural alterations. DIRT may represent not only transient edema but possibly also alterations in the laminar organization of the inner retina, which could involve complex changes in neuronal organization and glial cell activity (11). This structural disorganization might contribute to what could be considered an “interruption of the visual conduction pathway,” potentially limiting functional visual recovery even when anatomical edema appears to resolve.

Similarly, EZ/ELM disruption might reflect changes at the level of photoreceptor integrity (12). The ellipsoid zone, containing mitochondria, appears important for photoreceptor energy metabolism, while the external limiting membrane seems to provide structural support (13). Disruption of these layers could indicate photoreceptor alterations that might not fully respond to anti-VEGF therapy alone.

Some recent evidence suggests that the potential predictive value of these structural biomarkers might extend beyond anti-VEGF therapy. Studies investigating dexamethasone intravitreal implants have similarly reported DIRT and EZ/ELM integrity as potentially relevant prognostic factors (10, 14). This consistency across different treatment modalities might indicate that retinal structural integrity could be a factor worth considering in DME management across different therapeutic approaches.

The limited predictive value observed for systemic inflammatory indices (NLR, SII, PLR) requires careful interpretation. While systemic inflammation is generally recognized as involved in DME pathogenesis, several factors might explain our findings (15–17). The blood-retinal barrier appears to create some separation between systemic and retinal inflammatory environments, potentially complicating the relationship between peripheral blood markers and intraretinal pathology. Additionally, local retinal inflammation seems to involve complex mechanisms including microglial activation and cellular senescence processes (18, 19), which might not be fully captured by systemic indices alone.

The emerging understanding of the “gut-eye axis” (20) further adds complexity to these relationships, as systemic inflammation in DME might originate from multiple sources. While this systemic inflammation likely contributes to DME pathogenesis, its multifaceted nature might partially explain why local OCT features that directly visualize retinal structure could provide useful predictive information that complements systemic indicators.

Our prediction model demonstrated reasonably good discriminative ability (AUC = 0.869) with what appears to be adequate specificity (0.893) for identifying patients who might benefit from alternative treatment approaches. This level of specificity could be clinically useful for minimizing the risk of inappropriately withholding anti-VEGF treatment from potential responders, though this requires further validation.

The model’s performance suggests that baseline OCT evaluation might usefully complement treatment planning. Patients identified as potentially having poorer response to anti-VEGF monotherapy might be considered for more intensive monitoring or alternative approaches, though such decisions would need to be made in the context of individual patient factors and clinical judgment.

Several important limitations should be considered when interpreting our findings. The single-center retrospective design and moderate sample size (*n* = 81) suggest that our findings should be validated in larger, more diverse populations before broader application can be considered. The homogeneous population source is particularly relevant given known ethnic variations in retinal structure (21, 22).

The early response assessment (1 month post-loading) represents a practical timepoint for initial assessment but cannot fully capture longer-term response patterns. The clinical utility of early prediction needs to be balanced against the understanding that treatment response may evolve over time.

The comparison between systemic indices and OCT features involves methodological considerations that warrant attention. The observed differences in predictive performance might reflect variations in measurement precision rather than fundamental biological differences. Systemic indicators might require different methodologies or larger samples to demonstrate their potential predictive value.

Although we employed bootstrap validation, the use of stepwise regression with multiple predictors in a limited sample suggests that our findings should be considered preliminary rather than definitive. Finally, our model incorporates only baseline characteristics without considering how treatment responses might evolve over time.

Based on our findings, several research directions might be worth pursuing. Validation in larger multi-center prospective cohorts would help establish the generalizability of our observations. Exploring automated quantification of OCT features using computational methods might enhance reproducibility and clinical applicability. Investigating whether baseline OCT features might inform treatment selection between different anti-VEGF agents or combination therapies represents an interesting direction for future study.

Most importantly, interventional studies would be needed to determine whether modifying treatment based on predictive models actually improves patient outcomes. The potential clinical value of our model would be better understood through comparative effectiveness studies.

## 5 Conclusion

This comparative analysis suggests that retinal structural integrity assessed by OCT—particularly DIRT and EZ/ELM status—may provide useful predictive information for short-term anti-VEGF response in DME. The developed prediction model shows promising discriminative performance but requires validation in larger studies. Our findings tentatively support the integration of detailed OCT assessment into DME management while emphasizing the need for cautious interpretation of predictive information. The potential to identify patients who might respond differently to anti-VEGF therapy represents an area worthy of further investigation, though additional research is needed to understand how to best use this information in clinical practice.

## Data Availability

The raw data supporting the conclusions of this article will be made available by the authors, without undue reservation.

## References

[1] Berrocal MH Acaba LA Chenworth ML. Surgical innovations in the treatment of diabetic macular edema and diabetic retinopathy. Curr Diab Rep. (2019) 19:106. doi: 10.1007/s11892-019-1210-x, 31529405

[2] Cheema AA Cheema HR. Diabetic macular edema management: a review of anti-vascular endothelial growth factor (VEGF) therapies. Cureus. (2024) 16:e52676. doi: 10.7759/cureus.52676, 38264181 PMC10804209

[3] Ergin E Dascalu AM Stana D Tribus LC Arsene AL Nedea MI . Predictive role of complete blood count-derived inflammation indices and optical coherence tomography biomarkers for early response to Intravitreal anti-VEGF in diabetic macular edema. Biomedicine. (2025) 13:1308. doi: 10.3390/biomedicines13061308, 40564027 PMC12189659

[4] Amoaku WM Chakravarthy U Gale R Gavin M Ghanchi F Gibson J . Defining response to anti-VEGF therapies in neovascular AMD. Eye (Lond). (2015) 29:721–31. doi: 10.1038/eye.2015.48, 25882328 PMC4469673

[5] Gu W Wang M Li Z Xu T. The association between peripheral blood inflammatory markers and anti-VEGF treatment response in patients with type 2 diabetic macular edema. Front Med (Lausanne). (2025) 12:1653753. doi: 10.3389/fmed.2025.1653753, 41179902 PMC12571745

[6] Jin X Yang X Xu Y Liang J Liu C Guo Q . Differential correlation between time in range and eGFR or albuminuria in type 2 diabetes. Diabetol Metab Syndr. (2023) 15:92. doi: 10.1186/s13098-023-01071-4, 37386515 PMC10311716

[7] Lu W Xiao K Zhang X Wang Y Chen W Wang X . A machine learning model for predicting anatomical response to anti-VEGF therapy in diabetic macular edema. Front Cell Dev Biol. (2025) 13:1603958. doi: 10.3389/fcell.2025.1603958, 40519258 PMC12162914

[8] Koc F Güven YZ Egrilmez D Aydın E. Optical coherence tomography biomarkers in bilateral diabetic macular edema patients with asymmetric anti-VEGF response. Semin Ophthalmol. (2021) 36:444–51. doi: 10.1080/08820538.2021.1907423, 33780313

[9] Dou N Yu S Tsui CK Yang B Lin J Lu X . Choroidal vascularity index as a biomarker for visual response to Antivascular endothelial growth factor treatment in diabetic macular edema. J Diabetes Res. (2021) 2021:3033219. doi: 10.1155/2021/3033219, 34869776 PMC8642029

[10] Oliverio GW Meduri A Brancati VU Ingrande I De Luca L Raimondo ED . Clinical and optical coherence tomography biomarkers as prognostic factors in dexamethasone intravitreal implant for diabetic macular edema. Eur J Ophthalmol. (2024) 34:1810–8. doi: 10.1177/11206721241235242, 38384119

[11] Zhou L Xu Z Lu H Cho H Xie Y Lee G . Suppression of inner blood-retinal barrier breakdown and pathogenic Müller glia activation in ischemia retinopathy by myeloid cell depletion. J Neuroinflammation. (2024) 21:210. doi: 10.1186/s12974-024-03190-9, 39182142 PMC11344463

[12] Lee H Ji B Chung H Kim HC. Correlation between optical coherence tomographic HYPERREFLECTIVE foci and visual outcomes after anti-VEGF treatment in NEOVASCULAR age-related macular degeneration and POLYPOIDAL CHOROIDAL vasculopathy. Retina. (2016) 36:465–75. doi: 10.1097/iae.0000000000000645, 26076214

[13] Fu Z Sun Y Cakir B Tomita Y Huang S Wang Z . Targeting neurovascular interaction in retinal disorders. Int J Mol Sci. (2020) 21:1503. doi: 10.3390/ijms21041503, 32098361 PMC7073081

[14] Meduri A De Luca L Oliverio GW Mancini M Minutoli L Silvagno F . DEXAMETHASONE INTRAVITREAL INJECTION IN DIABETIC PATIENTS UNDERGOING CATARACT SURGERY: an updated literature review. Retina. (2025) 45:1030–42. doi: 10.1097/iae.0000000000004381, 39787414

[15] Zhang J Zhang J Zhang C Zhang J Gu L Luo D . Diabetic macular edema: current understanding, molecular mechanisms and therapeutic implications. Cells. (2022) 11:3362. doi: 10.3390/cells11213362, 36359761 PMC9655436

[16] Urias EA Urias GA Monickaraj F McGuire P Das A. Novel therapeutic targets in diabetic macular edema: beyond VEGF. Vis Res. (2017) 139:221–7. doi: 10.1016/j.visres.2017.06.015, 28993218

[17] Lai D Wu Y Shao C Qiu Q. The role of Müller cells in diabetic macular edema. Invest Ophthalmol Vis Sci. (2023) 64:8. doi: 10.1167/iovs.64.10.8, 37418272 PMC10337800

[18] Xu H Chen M Forrester JV. Para-inflammation in the aging retina. Prog Retin Eye Res. (2009) 28:348–68. doi: 10.1016/j.preteyeres.2009.06.001, 19560552

[19] Kawanaka N Taylor AW. Localized retinal neuropeptide regulation of macrophage and microglial cell functionality. J Neuroimmunol. (2011) 232:17–25. doi: 10.1016/j.jneuroim.2010.09.025, 20965575 PMC3030990

[20] Zysset-Burri DC Morandi S Herzog EL Berger LE Zinkernagel MS. The role of the gut microbiome in eye diseases. Prog Retin Eye Res. (2023) 92:101117. doi: 10.1016/j.preteyeres.2022.101117, 36075807

[21] Al-Zamil WM Al-Zwaidi FM Yassin SA. Macular thickness in healthy Saudi adults. A spectral-domain optical coherence tomography study. Saudi Med J. (2017) 38:63–9. doi: 10.15537/smj.2017.1.17565, 28042632 PMC5278067

[22] García-Franco R Méndez-Marín D García-Roa M Ramirez-Neria P Valera-Cornejo D Lansingh VC. Central macular thickness in a healthy Mexican population using Huvitz optical coherence tomography. Clin Ophthalmol. (2020) 14:3931–40. doi: 10.2147/opth.S272431, 33235432 PMC7680185

